# Choroidal Rift: A New OCT Finding in Eyes with Central Serous Chorioretinopathy

**DOI:** 10.3390/jcm9072260

**Published:** 2020-07-16

**Authors:** Marco Battista, Enrico Borrelli, Chiara Veronese, Francesco Gelormini, Riccardo Sacconi, Lea Querques, Francesco Prascina, Giovanna Vella, Antonio P Ciardella, Francesco Bandello, Giuseppe Querques

**Affiliations:** 1 Department of Ophthalmology, University Vita-Salute, IRCCS Ospedale San Raffaele, Via Olgettina, 58, 20132 Milano, Italy; marco.battista91@gmail.com (M.B.); borrelli.enrico@hsr.it (E.B.); francesco.gelormini@hotmail.it (F.G.); ric.sacconi@gmail.com (R.S.); querques.lea@hsr.it (L.Q.); francescoprascina@libero.it (F.P.); giovanna.vella28@gmail.com (G.V.); bandello.francesco@hsr.it (F.B.); 2 Ophthalmology Unit, S. Orsola-Malpighi Hospital, University of Bologna, Via Pelagio Palagi 9, 40138 Bologna, Italy; chiara.veronese@aosp.bo.it (C.V.); antonio.ciardella@aosp.bo.it (A.P.C.); 3 Ophthalmology, Department of Surgical, Medical, Molecular Pathology and of Critical Area, University of Pisa, Lungarno Antonio Pacinotti, 43, 56126 Pisa, Italy

**Keywords:** choroidal rift, optical coherence tomography, central serous chorioretinopathy

## Abstract

Central serous chorioretinopathy (CSC) is a complex and not entirely understood retinal disease. The aim of our research was to describe a novel optical coherence tomography (OCT) finding named “choroidal rift”, which may be identified in the choroid of eyes with CSC. We collected data from 357 patients (488 eyes) with CSC who had structural OCT and OCT angiography (OCTA) scans obtained. Choroidal rifts were identified as polygonal (and not round-shaped) hyporeflective lesions without hyperreflective margins. Choroidal rifts had to be characterized by a size superior to that of the largest choroidal vessel. Finally, hyporeflective lesions were graded as choroidal rifts only if these lesions had a main development perpendicular to the retinal pigment epithelium. OCT analysis allowed the identification of choroidal rifts in ten eyes from nine patients, all with chronic CSC, with an estimated prevalence rate of 2.1%. In three out of ten cases with choroidal rifts, these lesions spanned all the choroidal layers. In the remaining cases, choroidal rifts only partially spanned the choroidal thickness. In OCTA, choroidal rifts were characterized by the absence of flow. Combining structural OCT and OCTA information, we hypothesized that choroidal rifts may represent interruptions of the choroidal stroma in correspondence of fragile regions (in between expanded larger-sized choroidal vessels). Choroidal rift represents a novel OCT feature, which may characterize eyes with chronic CSC and may have a role in the development of irreversible chorio-retinal changes.

## 1. Introduction

Central serous chorioretinopathy (CSC) is a common chorioretinal disease included in the pachychoroid disease spectrum [1]. This disorder may be characterized by the accumulation of fluid in the subretinal and/or sub-retinal pigment epithelium (RPE) space [2]. The subretinal fluid (SRF) collection may cause photoreceptors’ dysfunction, this eventually resulting in visual symptoms such as blurred vision, metamorphopsia, micropsia and reduced contrast sensitivity [3].

Although CSC pathogenesis is multifactorial and not completely understood, the combination of a dysfunctional choroid, which is swollen and hyperpermeable, with an impaired RPE is widely considered as the primum movens eventually resulting in blood-retinal barrier malfunction and SRF accumulation. Furthermore, elevated levels of corticosteroids and/or catecholamine in the blood were demonstrated to be associated with dysfunction and ischemia of the choroidal capillaries, the latter process associated with a compensatory hyperpermeability and leakage of the sparing choroidal vessels [4]. Alternatively, an expansion of the larger-sized choroidal vessels may cause a choriocapillaris (CC) ischemia [5].

Optical coherence tomography (OCT) and OCT angiography (OCTA) technologies have significantly improved the choroidal characterization in eyes with CSC [6]. Importantly, en face OCT imaging displayed that areas of choroidal thickening in CSC eyes may be frequently topographically associated with pathologically dilated veins of the Haller’s layer (or “pachyvessels”) [5]. Notably, OCTA analysis granted the assessment of the choriocapillaris (CC) in eyes with pachychoroid disease, including CSC eyes [7,8]. In the latter studies, the authors demonstrated that the pathological choroidal thickening may be associated with inner choroidal (or CC) ischemia.

Previous important studies employing OCT technology demonstrated that CSC eyes may be characterized by two peculiar hyporeflective regions within the choroidal volume: (i) choroidal caverns and (ii) choroidal loculations. While the latter were described as hyporeflective regions between the outer segment of choroid and sclera [9], the former were described as irregularly small round-shaped hyporeflective spaces within the choroid [10,11,12].

We recently noted the presence of large choroidal hyporeflective spaces in eyes with CSC, which have peculiar characteristics in comparison with both choroidal caverns and loculations. Because of their anatomical characteristics as revealed by structural OCT images, these alterations were named “choroidal rifts”. Therefore, the aim of this study was to provide an integrated description of choroidal rifts and to assess their prevalence in eyes with CSC.

## 2. Methods

### 2.1. Study Participants

In our retrospective study, subjects 18 years of age and older with history or evidence of CSC in at least one eye were identified from the medical records of the “Medical Retina & Imaging” unit at the San Raffaele Hospital, Milan. The study adhered to the 1964 Helsinki declaration and its later amendments. Informed consent was obtained from all individual participants included in the study and it was approved by the Local Institutional Review Board (IRB).

All patients were imaged with the OCT Spectralis (Heidelberg Engineering, Heidelberg, Germany) and PLEX Elite 9000 (Carl Zeiss Meditec Inc., Dublin, CA, USA) devices between January 2018 and December 2019. All patients received a complete ophthalmologic examination.

The authors in this study identified patients with CSC using the clinical and imaging criteria previously described [13]. Exclusion criteria included (i) history or evidence of other retinal disorders and (ii) poor-quality scans or images with segmentation errors. In patients with more OCT and OCTA scans obtained at consecutive visits, we included scans obtained at the latest visit.

### 2.2. Study Protocol

The OCT and OCTA images of each included eye were reviewed by two graders (MB and EB) to identify the presence of choroidal rifts. Choroidal rifts were identified as polygonal (and not round-shaped) hyporeflective lesions (in contrast to choroidal caverns) without hyperreflective margins. Choroidal rifts had to be characterized by a size superior to that of the largest choroidal vessels. Finally, hyporeflective lesions were graded as choroidal rifts only if these lesions had a development perpendicular to the RPE (in contrast to choroidal loculations).

Each grader performed the analysis and interpretation separately. Graders later met to compare the level of agreement and disagreements were solved by further discussion and open settlement to provide a single assessment for each case.

Finally, on OCTA images, the two graders identified and confirmed the related regions of blood flow in the choroid.

### 2.3. Statistical Analysis

The analysis included descriptive statistics (using Microsoft Office Excel software; version 14.0, 2010, Redmond, WA, USA) for demographics and main clinical data and qualitative descriptions of the imaging characteristics.

The incidence rate was calculated by the number of CSC cases with choroidal rifts identified divided by the total number of patients.

## 3. Results

Our study cohort included 357 patients (488 eyes of 277 males and 80 females; the mean ± SD age was 53.2 ± 10.9 years), all with chronic CSC (persistent sub-retinal fluid for more than 6 months). Within this study cohort, OCT grading allowed the identification of choroidal rifts in ten eyes from nine patients (seven males and two females; the mean ± SD age was 55.9 ± 8.5 years) with an estimated prevalence rate of 2.1%.

No patient in this subgroup had a clinical history of diabetes or uncontrolled systemic hypertension. Within patients with choroidal rifts, three out of nine patients were previously treated with photodynamic therapy (PDT), while five out of nine patients had treatment with eplerenone. None of our patients had anti-vascular endothelial growth factor (VEGF) therapy at the time of inclusion.

**Figure 1:**
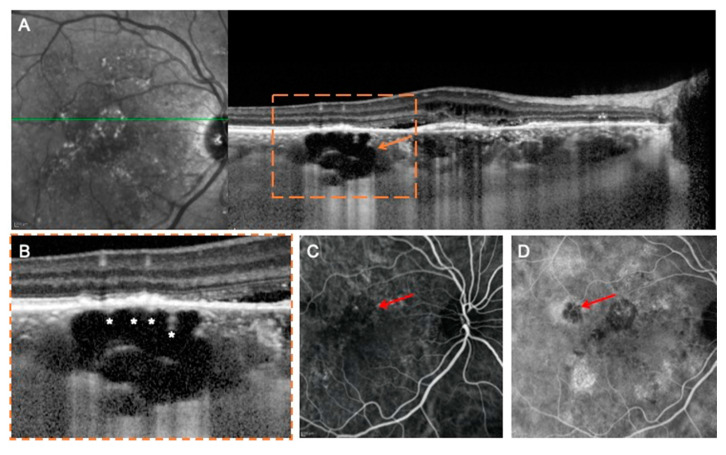
Multimodal imaging of a choroidal rift in a case of chronic central serous chorioretinopathy (CSC). (**A**) Optical coherence tomography (OCT) B-scan and relative IR image with the over-imposed line of scanning demonstrating the presence of a large choroidal rift (orange arrow) crossing all the choroidal layers and seen as a grossly empty prismatic space. A magnified visualization of this region (**B**) revealed the presence of hyperreflective vertical filiform-shaped structures (white asterisks) within choroidal rifts, which were speculated to represent the remnant of intercapillary pillars. Early (**C**) and intermediate (**D**) indocyanine green angiography (ICGA) images demonstrate that the region occupied by the choroidal rift (highlighted with the red arrows) is characterized by a reduced fluorescence because of an absent/reduced choroidal filling.

**Figure 2:**
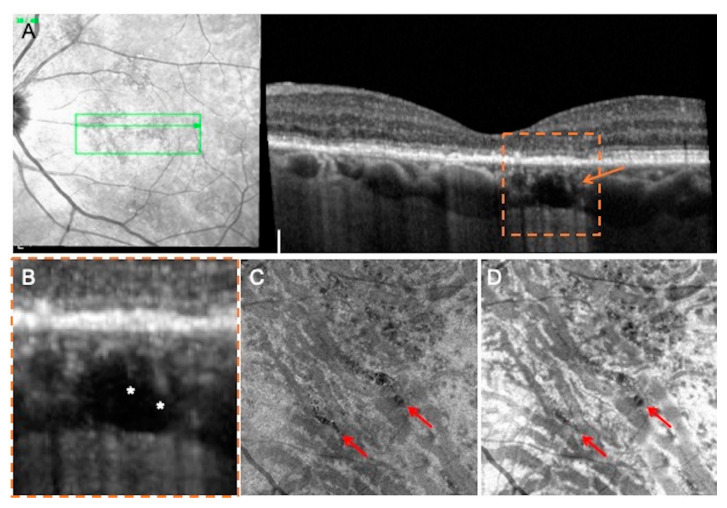
Multimodal imaging of a patient affected by chronic CSC and presenting a choroidal rift. (**A**) OCT B-scan and relative IR image shows the presence of a choroidal rift (orange arrow) crossing the outer choroidal layers. (**A**) magnified visualization of this region; (**B**) reveals the presence of hyperreflective vertical filiform-shaped structures (white asterisks). En face angiographic (**C**) and structural (**D**) OCT images revealed that choroidal rifts (highlighted with the red arrows) seem to be interruptions of the choroidal stroma in correspondence of fragile regions (in between major-sized choroidal vessels).

The general appearances of these hyporeflective lesions are shown in Figure 1 and Figure 2. In three out of ten eyes with choroidal rifts, these lesions spanned all the choroidal layers, including the choriocapillaris (Figure 1). In the remaining cases, choroidal rifts only partially spanned the choroidal thickness (Figure 2). In six cases (Figure 1 and Figure 2), hyperreflective vertical filiform-shaped structures were identified within choroidal rifts and were speculated to represent intercapillary pillars of the choroidal vessels, which remained patulous within choroidal rifts. On OCT images, choroidal rifts, which per definition had an irregular shape without hyperreflective margins and they had dimensions superior to the largest choroidal vessels, presented a mean ± SD dimension of 215 ± 78.6 and 589 ± 374.7 μm on the vertical and horizontal diameters, respectively.

In the evaluation of the OCTA images, choroidal rifts were characterized by absence of flow. More importantly, in the combined evaluation of en face structural and angiographic OCT images, choroidal rifts seemed to be interruptions of the choroidal stroma in correspondence of fragile regions (in between expanded larger-sized choroidal vessels) (Figure 2).

## 4. Discussion

In this cross-sectional study, we identified and described a novel structural OCT finding in the choroid of patients with chronic CSC, which was named “choroidal rift” because of its anatomical characteristics. Furthermore, using OCTA, we demonstrated the absence of flow inside these choroidal lesions.

Several notable previous studies employing OCT identified peculiar choroidal spaces in normal and affected eyes. Querques et al. [10] first identified the presence of choroidal caverns in eyes with geographic atrophy and with an estimated prevalence of 12.5%. These lesions were described as gaping hyporeflective cavities within the choroid, which were easily distinguishable from choroidal vessels as the former are typically empty, angular and without hyperreflective borders. Successively, Sakurada and colleagues [12] identified choroidal caverns in eyes with CSC. Interestingly, in the latter study, the authors highlighted a topographical correlation between the presence of choroidal caverns on structural OCT and choroidal vascular hyper-permeability as evaluated with indocyanine green angiography (ICGA) [12]. Recently, Dolz-Marco et al. [14] provided a fully integrated description of choroidal caverns in healthy and pathologic eyes, including eyes with a pachychoroid spectrum of diseases. In the latter study, two distinct groups of eyes were enrolled for histopathological and imaging assessment of the choroidal caverns. Based on their results, the authors concluded that Friedman’s lipid globules were the histologic correlate of choroidal caverns. However, the correspondence between choroidal caverns and lipid globules is still a matter of debate [15], assuming that choroidal caverns are mostly characterized by late hyperfluorescence on ICGA images and a lipid (hydrophobic) composition of these lesions could not exhibit this kind of ICGA peculiarity [16].

Choroidal loculations represent another OCT finding that may be detected in eyes with CSC [9]. Choroidal loculations were described as hyporeflective spaces located in the outer choroid and with an angular inner border and a size superior to that of the larger choroidal vessels. These choroidal lesions were speculated to be induced by hyperpermeability in the peripheral choroid, where the attachment to the sclera is weaker [9].

**Figure 3:**
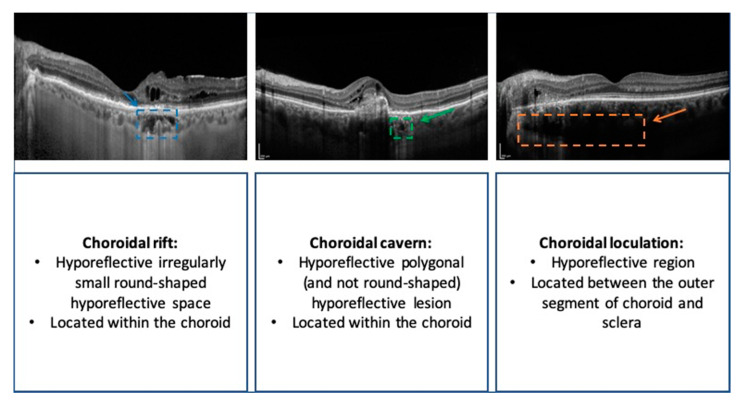
Schematic representation of the different choroidal findings that may be seen in patients with chronic CSC.

We have added to the literature by reporting a new OCT finding identified in the choroid of patients with chronic CSC (see Figure 3 for a comparison among choroidal findings in CSC). Choroidal rifts appear as hyporeflective empty spaces developed in the context of a thickened choroid. Contrary to choroidal caverns, choroidal rifts are not characterized by an irregularly small round shape as they appear as asymmetrical multi-lobulated hyporeflective structures with an extension that can also involve the whole choroid. Additionally, in contrast to choroidal caverns and similarly to choroidal loculations, choroidal rifts were larger than the largest choroidal vessel. However, while choroidal loculations have a predominant horizontal growth, choroidal rifts mainly develop perpendicularly to the RPE as they seem to interrupt the choroidal volume.

**Figure 4:**
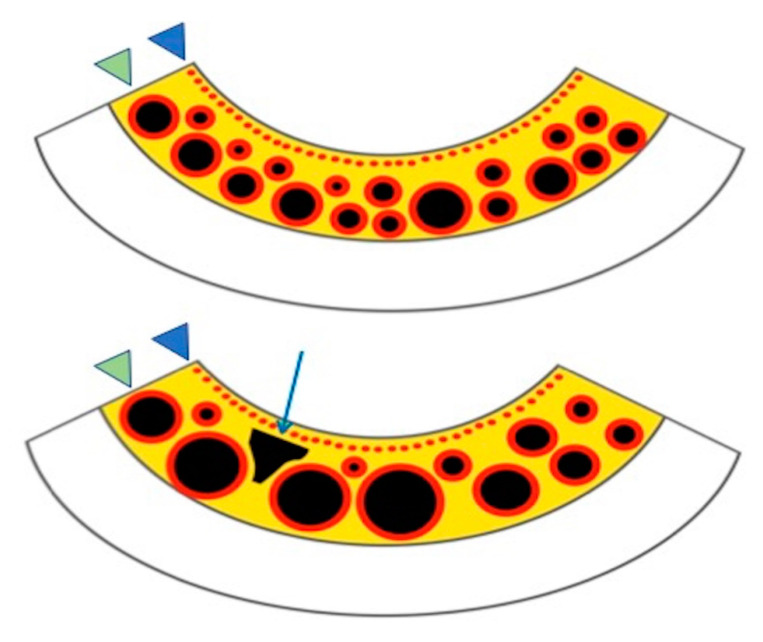
Drawing showing the possible pattern of choroidal rift formation in eyes with central serous chorioretinopathy. A schematic representation of a normal (top) and thickened choroid in CSC (bottom) showing the choriocapillaris (small red circles highlighted with the blue head arrows) and Sattler’s and Haller’s layers (red and black circles highlighted with the green head arrows) within choroidal stroma (yellow regions). An expansion of the choroid occurring in CSC is accompanied by a reduction in both choriocapillaris and larger large-sized choroidal vessels. The latter event, along with an antero-posterior stretching of the choroidal volume, may cause a mechanical stress to the choroidal stroma resulting in the development of a choroidal rift (highlighted with the blue arrow).

We may hypothesize that choroidal rifts represent a further step in the vascular remodeling of choroidal vasculature in eyes with CSC. Choroidal caverns were speculated to represent nonperfused ghost vessels [10] or, alternatively, they may constitute regions of fractures of the choroidal stroma [15]. Similarly, we may speculate that a further antero-posterior stretching of the choroidal tissue might result in the formation of these choroidal rifts that could represent empty spaces with neither vessels nor stroma (Figure 4). Our analysis on en face OCT and OCTA images further corroborated this hypothesis, as these images revealed that choroidal rifts colocalize with the fragile regions of the choroidal stroma (in between expanded larger-sized choroidal vessels) and seem to represent breaks of the stretched connective tissue of the stroma.

Choroidal rifts were only detected in eyes affected by a chronic stage of CSC, hence could be considered as a sign of persistency of the disease contributing to the morpho-functional severity of the chorio-retinal involvement. However, it is important to highlight that, similarly to choroidal caverns that were detected in different disorders, for choroidal rifts we were not able to exclude that these features were actually present in other chorioretinal affections. Future studies will clarify this aspect.

The main limitation of this paper is that the data generated in our study are the result of analyzing OCT images, not by direct histologic examination. Other limitations of our study include the retrospective nature of its analysis and the limited number of cases of choroidal rifts identified. Moreover, patients enrolled had been treated with different therapies, which further reduced the cohort homogeneity. However, it must be noted that some of our patients (three out of nine) were treatment-naïve at the moment of study inclusion. Therefore, we might suppose that this structural OCT finding was not associated with the treatment, but rather was dependent on the disease evolution.

In conclusion, this study identified a new OCT finding located in the choroid of eyes with chronic CSC. This novel finding was named “choroidal rift” because of its characteristics on the OCT images. Future longitudinal studies with larger cohorts would be needed to elucidate mechanisms leading to these lesions and their role in the development of the chronicity of the disease as well as in the development of irreversible retinal changes.
